# Familial co-segregation and the emerging role of long-read sequencing to re-classify variants of uncertain significance in inherited retinal diseases

**DOI:** 10.1038/s41525-023-00366-9

**Published:** 2023-08-10

**Authors:** Pankhuri Gupta, Kenji Nakamichi, Alyssa C. Bonnell, Ryan Yanagihara, Nick Radulovich, Fuki M. Hisama, Jennifer R. Chao, Debarshi Mustafi

**Affiliations:** 1 Division of Medical Genetics, Department of Medicine, University of Washington, Seattle, WA 98109 USA; 2 Department of Ophthalmology and Roger and Karalis Johnson Retina Center, University of Washington, Seattle, WA 98109 USA; 3 Division of Ophthalmology, Seattle Children’s Hospital, Seattle, WA 98105 USA; 4 Brotman Baty Institute for Precision Medicine, Seattle, WA 98195 USA

**Keywords:** Hereditary eye disease, Next-generation sequencing

## Abstract

Phasing genetic variants is essential in determining those that are potentially disease-causing. In autosomal recessive inherited retinal diseases (IRDs), reclassification of variants of uncertain significance (VUS) can provide a genetic diagnosis in indeterminate compound heterozygote cases. We report four cases in which familial co-segregation demonstrated a VUS resided in *trans* to a known pathogenic variant, which in concert with other supporting criteria, led to the reclassification of the VUS to likely pathogenic, thereby providing a genetic diagnosis in each case. We also demonstrate in a simplex patient without access to family members for co-segregation analysis that targeted long-read sequencing can provide haplotagged variant calling. This can elucidate if variants reside in *trans* and provide phase of genetic variants from the proband alone without parental testing. This emerging method can alleviate the bottleneck of haplotype analysis in cases where genetic testing of family members is unfeasible to provide a complete genetic diagnosis.

## Introduction

Phasing genetic variation is critical for assigning causative variants in autosomal recessive (AR) diseases. Autosomal recessive is the most prevalent inheritance pattern^1^ for inherited retinal diseases (IRDs)^2^, which are a heterogeneous group of predominantly monogenic disorders with over 300 causative genes identified^3^. A catalog of genetic variations present in the human population from the Genome Aggregation Database (gnomAD)^4^ estimates that over 5 million people worldwide are expected to be affected with an autosomal recessive (AR) IRD, with nearly 3 billion healthy carriers^5^. Genetic diagnosis of IRDs can guide treatment decisions^6^, allow access to FDA-approved treatment^7^, and aid in patient selection of approved treatments in the future^8^. However, currently in 20–30% of AR IRD cases one or no disease-causing variants are identified from short-read exome sequencing^9,10^. In nearly 20% of cases, genetic testing reveals at least one variant of unknown pathogenicity, known as a variant of uncertain significance (VUS)^11^, which are non-diagnostic, but over time, may be reclassified as likely pathogenic or pathogenic^12^. Reclassification of a VUS can increase the molecular diagnosis rate of IRDs and provide patients and families with accurate recurrence risks.

Guidelines to define variant pathogenicity have been established by the American College of Medical Genetics (ACMG) that incorporate haplotype information, data from population databases (such as gnomAD), computational predictive programs, functional studies, co-segregation analysis, and allelic frequencies as criteria for variant classification. One key step to reclassifying VUSs is phasing patients’ genetic variants^13^. This is especially helpful in AR cases where one pathogenic variant and one VUS are identified in a disease gene that fits the clinical phenotype. Next-generation sequencing (NGS) approaches commonly used to genetically diagnose the disease are generally unable to decipher whether variants are in the *cis* or *trans* configuration as the short-read lengths usually only span a single variant. Thus, in order to demonstrate two variants reside in *trans*, familial co-segregation analysis is carried out. Genetic testing of the patient’s parents can then resolve the chromosomal arrangement of the identified variants^14^. If genetic testing reveals that one pathogenic variant was paternally inherited and the other was maternally inherited, it can be inferred that these variants reside in *trans* on separate chromosomes. Haplotype analysis demonstrating variants residing in *trans* can provide additional evidence to support pathogenicity for a VUS. In cases where a patient’s parents are unable to be tested, other family members may undergo genetic sequencing to demonstrate variants reside in *trans*.

Emerging long-read sequencing technology now allows the partitioning of sequencing reads into two parental genome datasets, genetically phasing an individual genome without the need for familial DNA analysis^15^. This is beneficial when a patient’s family members are unable to be tested, and/or access to a patient’s family member is limited. In these cases, long-read sequencing for haplotype determination offers the potential for efficient genetic phasing in the future and provides a cost-effective solution without the need for parental retesting. This technology is already being implemented in other fields of medicine, and there is a need for this technology in ophthalmic genetics. In this paper, we use traditional familial co-segregation analysis for four cases to decipher whether variants reside in *cis* or *trans*, which allows the reclassification of four VUSs into likely pathogenic/pathogenic (Table 1). We also report a fifth case in which in the absence of familial data long-read sequencing provides haplotype information with potential disease-causing variants in *trans* that results in reclassification of the VUS to likely pathogenic.

**Table 1:** Patient demographics and novel genetic variants identified after next-generation sequencing.

Case number	Retinal disease	Gene	Pathogenic variant (coordinates in GRCh38)	gnomAD database allele frequency	Variant of uncertain significance	gnomAD database allele frequency	VUS variant type	Variant pathogenicity predictions	ACMG criteria met	VUS reclassification
1	Senior–Løken syndrome	*NPHP4*	c.133C>T; (p.Gln45Ter (Chr1: 5986157))	3.29e−5	c.517+50C>G (Chr1: 5967249	6.57e–6	Intronic/Splice	CADD score: 11 Splice AI: 0.90 (donor gain)	SP3, PM2, PM3, PP3	Likely pathogenic
2	Achromatopsia	*CNGA3*	c.1306C>T;p.Arg436Trp (Chr2: 98396476)	6.58e−5	c.1584G>C ; p.Val529Leu (Chr2: 98396754)	Not present	Missense	CADD score: 23.5	PM2, PM3, PM5	Likely pathogenic
3	Retinitis pigmentosa	*IMPG2*	Cc.3023-6_3030dup; p.Ala1011PhefsX2 (Chr3: 101232983)	3.94e−5	c.3423-7_3423-4delCTTT (Chr 3: 101229593)	1.64e−4	Intronic/Splice	CADD score: 12.5 Splice AI: 0.38 (acceptor loss)	SP3, PM2, PM3, PP3	Likely pathogenic
4	Retinitis pigmentosa	*IMPDH1*	None	–	c.942_944delGAA; p.Lys314del (chr7:128398545)	Not present	Inframe deletion	CADD score: 21.5 Splice AI: 0.01 (donor gain)	PS2, PM2, PM4, PP3	Likely pathogenic
5	Usher syndrome	*USH2A*	c.10820A>C; p.His3607Pro (Chr1:215779962)	1.97e−5	c.14996C>T; Thr4999Ala (Chr1:215639212).	Not present	Missense	CADD score: 24.2	PM2, PM3, PM5	Likely pathogenic

## Results

### Familial co-segregation analysis reveals chromosomal origin of pathogenic variants in a compound heterozygote

**Fig. 1:**
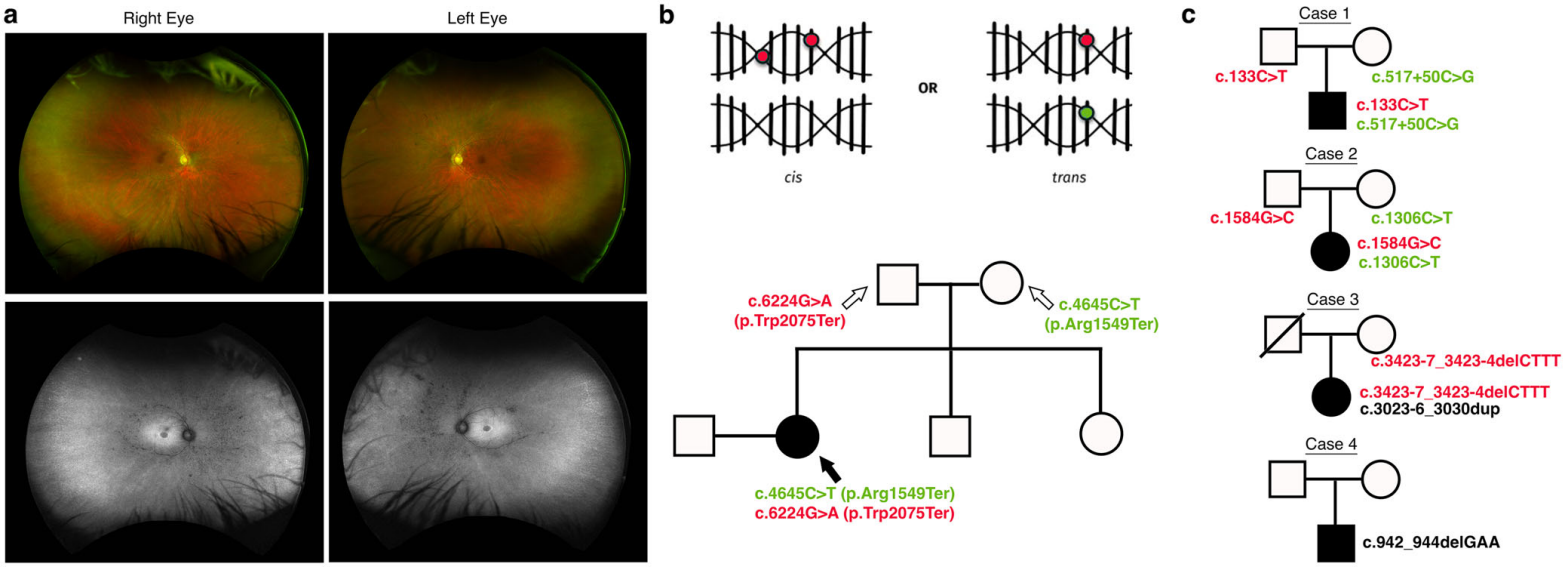
Familial co-segregation analysis demonstrates chromosomal architecture of identified variants. **a** Widefield color fundus photographs reveal rare bone spicule lesions in the far nasal periphery which are more prominent on autofluorescence images in a patient with clinical features of Usher syndrome. **b** Variants can be identified from NGS but the short-read lengths limit the ability of NGS to determine if said variants reside in *cis* or in *trans*. Familial co-segregation analysis can demonstrate if variants reside in *trans*, as in this case, where the two pathogenic variants in *USH2A* identified in the proband (black filled-in circle) can be traced from her parents who each carry one of the two variants, with the proband’s father harboring the c.6224G>A (p.Trp2075Ter) variant and the proband’s mother harboring the c.4645C>T(p.Arg1549Ter) variant. **c** In Cases 1–3, the variants in each proband were demonstrated to be in *trans* with familial co-segregation analysis, whereas in Case 4 the variants were confirmed to arise de novo after familial co-segregation testing. Paternally inherited variants are denoted in red whereas those denoted in green were maternally inherited.

To identify if variants identified from short-read sequencing studies reside in *trans* using familial co-segregation in an AR IRD, we present a 31-year-old female with progressive nyctalopia, visual field loss in both eyes and hearing loss since childhood. Fundoscopic examination revealed peripheral retinal pigment epithelium loss and bone spicules bilaterally (Fig. 1a). In conjunction with the patient’s reported hearing loss, there was a clinical concern for the syndromic IRD, Usher syndrome. Panel-based NGS identified two known pathogenic variants in the Usherin (*USH2A*) gene, c.6224G>A (p.Trp2075Ter) and c.4645C>T (p.Arg1549Ter). Parental testing revealed that the c.6224G>A (p.Trp2075Ter) variant was paternally inherited and the c.4645C>T (p.Arg1549Ter) variant was maternally inherited, confirming that these two variants were inherited in *trans* (Fig. 1b) to explain the phenotypic features in this case.

### Familial co-segregation analysis with parental testing demonstrates chromosomal phase information of variants of unknown significance

We present four cases in which parental testing was available to examine the chromosomal architecture of identified variants. In each case, the patient presented to the ophthalmology clinic with vision changes and was suspected of having an IRD based on clinical examination. Panel-based NGS for Cases 1–3 revealed a pathogenic variant as well as a VUS in a gene known to cause an AR IRD. For Case 4, a single VUS was noted in a gene known to cause an autosomal dominant IRD. Parental testing in each case confirmed that the VUS was either detected in *trans* in AR cases with a pathogenic variant or arose de novo in the autosomal dominant case. The moderate evidence from phase information in addition to other strong and moderate criteria detailed for each case, allowed the reclassification of each VUS as likely pathogenic (Table 1), and thus provide a definitive molecular diagnosis for their clinical presentations.

### Case 1

Case 1 details a 21-year-old male with concern for Senior–Løken syndrome given his ocular presentation consistent with retinitis pigmentosa (RP) and history of juvenile nephronophthisis. On genetic testing, he was found to have two variants in the Nephrocystin 4 (*NPHP4*) gene, a known-pathogenic variant, and a VUS. The pathogenic variant, c.133C>T (p.Gln45Ter), was found to be paternally inherited and the intronic VUS, c.517+50C>G, was maternally inherited, which demonstrated that the variants reside in *trans* (PM3). The VUS was present in the gnomAD database at a rare allelic frequency of 6.57e−6 (PM2), and predictive tools of pathogenicity such as CADD and SpliceAI indicated this would result in a donor gain (Table 1) leading to aberrant splicing of the gene (PP3). The VUS in Case 1 had previously been confirmed to cause aberrant splicing by PCR and Sanger sequencing in a fibroblast cell line from a patient known to have the same variant^16^ (PS3). Thus, 1 strong, 2 moderate, and 1 supporting criterion allow reclassification of this VUS as likely pathogenic.

### Case 2

Case 2 is a 4-year-old female who presented due to decreased vision and photophobia with a negative family history of eye diseases. An electroretinogram demonstrated an absent photopic response with an electronegative waveform in scotopic conditions concerning achromatopsia. Genetic testing revealed the presence of the most common pathogenic variant in Cyclic Nucleotide-Gated Channel, Alpha-3 (*CNGA3*), c.1306C>T (p.Arg436Trp). The second variant identified by genetic testing, c.1584G>C (p.Val529Leu), was a VUS. Parental testing revealed that each variant was inherited from each parent, confirming their presence in *trans* (PM3). The VUS was not present in the gnomAD database (PM2), but at that precise genomic location, another pathogenic missense variant c.1585G>A (p.Val529Met) was present. This provides moderate evidence for a missense change at an amino acid where a different missense change is pathogenic (PM5). Together, three moderate criteria presented allow the reclassification of this VUS as likely pathogenic.

### Case 3

In Case 3, a 35-year-old female was suspected of having retinal degeneration upon ophthalmic evaluation. NGS showed that there were two variants in the Interphotoreceptor Matrix Proteoglycan 2 (*IMPG2*) gene, which is known to cause AR RP. One variant was known to be pathogenic, c.3023–6_3030dup (p.Ala1011PhefsX2), and the other was a VUS, c.3423-7_3423-4delCTTT. In this case, only the mother was available for genetic testing because the father had passed away. The mother was found to harbor the VUS after targeted testing of the *IMPG2* gene. It was presumed the pathogenic variant was inherited from the father and thus the two variants segregate in *trans* (PM3). The VUS in this case was an intronic variant that was present in the gnomAD database at a rare allelic frequency of 1.64e−4 (PM2). Furthermore, through scores from predictive tools CADD and SpliceAI, the VUS was anticipated to have a deleterious effect on splicing (Table 1), resulting in aberrant protein formation through the loss of a splice acceptor region (PP3). Functional studies have demonstrated that this VUS affects the splice acceptor site and leads to a frameshift and premature termination of IMPG2 by utilizing a second splice site upstream in the same intron^17^ (PS3). Thus, 1 strong and 2 moderate criteria allow reclassification of this VUS as likely pathogenic.

### Case 4

Case 4 centers around a 22-year-old male whose clinical examination was consistent with RP with no family history of eye disease. Genetic testing in this case revealed a single VUS, c.942_944delGAA (p.Lys314del), in the Inosine Monophosphate Dehydrogenase 1 (*IMPDH1*) gene. Pathogenic variants in *IMPDH1* are known to cause autosomal dominant RP^18^. Familial testing of the *IMPDH1* gene revealed that neither parent carried the VUS, suggesting that the VUS arose de novo in this case (PS2). This variant was not present in the gnomAD database (PM2) and resulted in an in-frame deletion that changes the protein length (PM4) and is predicted to be deleterious by CADD and SpliceAI scores listed in Table 1 (PP3). Furthermore, there is evidence that missense variants resulting in change at the same amino acid site are pathogenic, as the c.942G>C (p.Lys314Asn) is noted on ClinVar to be pathogenic. Thus, strong criteria of an identified de novo variant confirmed by familial co-segregation along with 2 moderate and 1 supporting criteria allow reclassification of this VUS as likely pathogenic.

### Case 5: Targeted long-read sequencing allows for variant identification and haplotyping from proband alone in the absence of familial data

**Fig. 2:**
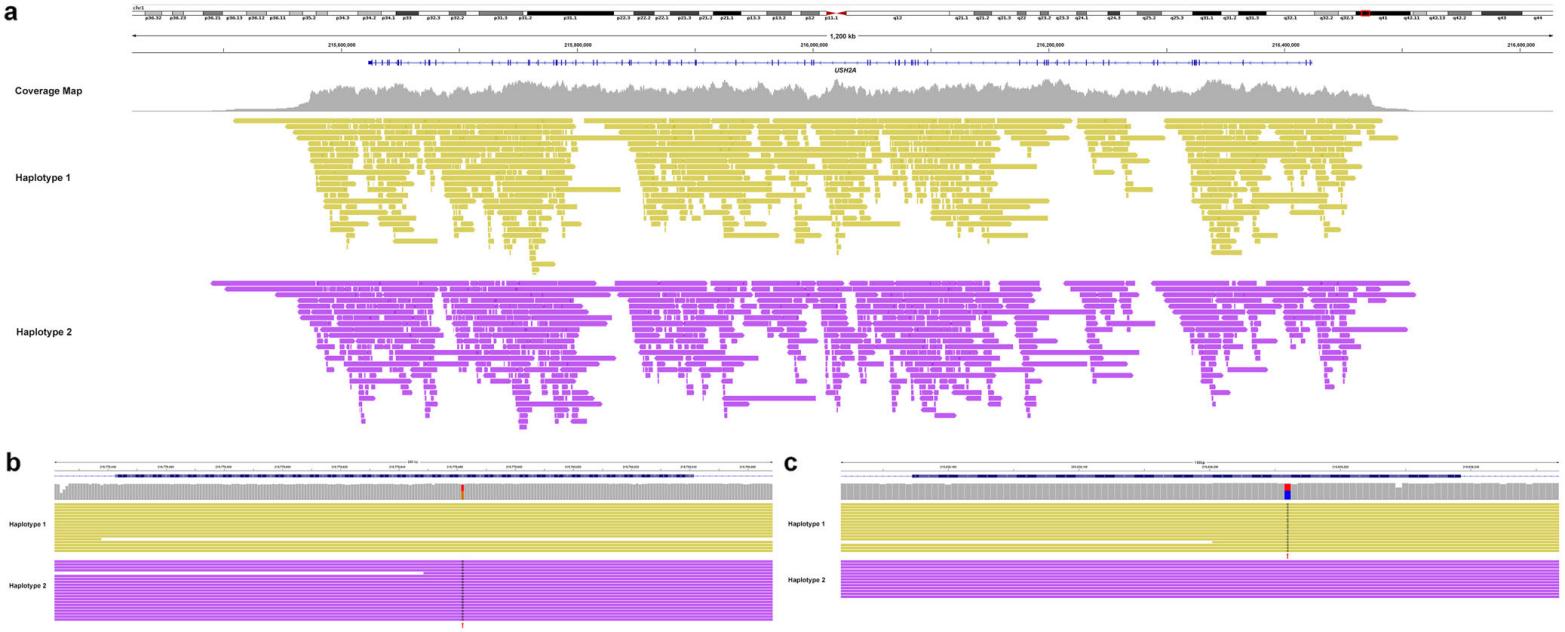
Long-read sequencing of Case 5 with Usher syndrome reveals that a pathologic variant, c.10820A>C (p.His3607Pro), and a variant of unknown significance (VUS), c.14996C>T (Thr4999Ala), in the *USH2A* gene, reside in *trans* in the absence of familial co-segregation analysis. **a** Targeted sequencing of the *USH2A* gene locus using Readfish adaptive technology revealed robust coverage of the *USH2A* locus with low base coverage outside of this selected genomic region. The depth of coverage obtained from targeted sequencing enrichment allowed for haplotyping. A closer examination of exons 55 and 69 shows the excellent base coverage in the region that the two variants reside. **b** The variant on exon 55 (c.10820A>CA) and **c** variant on exon 69 (c.14995A>G) are indicated by red arrows and are clearly found on opposite chromosomes in a *trans* configuration.

Genotyping data obtained from the DNA of parents or other family members is most important in computational phasing strategies^19,20^ but may not always be available. Case 5 details a 66-year-old male who presented with visual changes and hearing loss suspicious of Usher syndrome. Genetic testing revealed two variants in the *USH2A* gene. These included a known-pathogenic variant, c.10820A>C (p.His3607Pro), and a VUS, c.14995A>G (p.Thr4999Ala). The variants could not be phased by the short-read technology initially used for clinical panel-based exome sequencing. Parental testing was not possible in this case so targeted long-read sequencing of the *USH2A* gene locus was carried out on the Oxford Nanopore Technologies platform^21^. The long reads could be partitioned into two haplotypes and demonstrated that the 2 variants reside in *trans* (Fig. 2) (PM3). The VUS was not present in the gnomAD database (PM2) and this missense change at amino acid 4999 was in the same location as a different missense change c.14996C>T (p.Thr4999Ile) that has been determined to be pathogenic in ClinVar (PM5). Despite the absence of familial data, phasing of the variants in the absence of trio testing provided additional moderate criteria to reclassify the VUS as likely pathogenic.

## Discussion

The diagnosis of an IRD is based on clinical and familial findings, in which genetic testing plays an important role in predicting disease risks and outcomes. This is especially true for autosomal recessive IRDs in which confirmation of biallelic pathogenic variants with clinical suspicion of an IRD can potentially alter medical management and provide proper risk stratification for future generations. Pedigree-based haplotype inference can be carried out with family DNA samples to ascertain if a variant resides in *trans* autosomal recessive disorders, which provides moderate evidence in the classification of variants as likely pathogenic or pathogenic^22^. In this work, we detail three cases in which familial co-segregation established that a VUS resides in *trans* to a known pathogenic variant in an IRD-causing gene with AR inheritance. We also present a fourth case in which familial co-segregation demonstrated a VUS arose de novo in an autosomal dominant IRD-causing gene. Distinguishing disease-causing variants from variants of uncertain significance is a challenge in current clinical genetics. To establish the potential pathogenicity of these VUS we evaluated them according to the ACMG Standards and Guidelines for Sequence Variant Interpretation^22^. The rarity of a variant, assessed as low allelic frequency or absence from large human population databases, provides a criterion for further considering a variant as possibly pathogenic. Examination of the gnomAD database revealed that 2 out of the 4 cases had a VUS that was present at low allelic frequencies, with the VUS from *IMPG2* at the highest frequency of 1.64e−4. The other 2 were not present in the gnomAD database, out of which Case 2 had a different missense variant at the precise genomic location of the noted VUS (p.Val529Leu). This missense variant, identified to be pathogenic, was found in the gnomAD database (c.1585G>A (p.Val529Met)) at an allelic frequency 3.28e−5. Thus in the case of Case 2, in addition to the absence of the VUS from the gnomAD database, we also found evidence of a missense change at an amino acid residue in which a different missense change (p.Val529Met) has been deemed pathogenic. The VUS in Case 4 was not present in gnomAD data and examination of that region of *IMPDH1* reveals good genomic coverage, suggestive that this is not due to low coverage of a genomic region.

Statistical frameworks for variant frequency can filter variants to assess those that may be rare enough to cause disease^23^. Using this approach, and based on the prevalence and genetic and allelic heterogeneity of autosomal recessive IRDs, we estimate that variants with a frequency >3e−4 may be too common to be possibly pathogenic. However, there are gene-specific exceptions as evidenced by relatively common variants in the *ABCA4* gene causing AR IRD disease^24^. To this end, we find that all the VUS identified in this study that is present in the gnomAD database have allelic frequencies lower than the established 3e−4 cut-off and are rare enough to be considered as possibly pathogenic. Predictive algorithms such as PolyPhen-2, SIFT, CADD, and SpliceAI along with functional assays performed in the past provided additional supportive evidence of the potential pathogenicity of these rare VUS. These cumulative lines of evidence enabled the reclassification of these VUS as likely pathogenic for Cases 1–4.

Reclassification of a VUS as a likely pathogenic disease-causing variant can provide evidence of biallelic disease in AR IRDs and open up opportunities for patients to access disease-relevant clinical trials. In this report, we outline the delineation of a VUS in *CNGA3* in Case 2 as likely pathogenic which provides biallelic evidence of disease leading to the observed phenotypic features of achromatopsia. This new evidence would enable this patient to be enrolled in a clinical trial^25^. The reclassification of a VUS can take many years after initial genetic testing due to the availability of familial data to the assignment of haplotyped information, which can delay care delivery to these vulnerable patients. As outlined in this work, once a VUS is established to be inherited in *trans* to a pathogenic variant in an AR IRD, supporting evidence to reclassify the variant relies more on computational approaches and less reliant on the patient or familial sample input. One potential bottleneck to obtaining familial testing can be overcome by incorporating long-read sequencing for clinical haplotype analysis of only the patient in question. With current sequencing technologies, co-segregation analysis is the primary technique for haplotype reconstruction. Commercial laboratory short-read sequencing methods are usually limited because they can read only small sequence lengths at a time (up to 300 base pairs) and typically span a single variant. Another limitation arises with pedigree-based haplotype inference, which depends on the availability of familial DNA samples and cannot phase de novo variations that have arisen in the last generation. Applying emerging long-read sequencing technology to patients with an IRD can provide solutions to both of these limitations. Long-read sequencing can provide unique phasing information to determine the haplotype on which a variant occurs to resolve recessive disease-associated alleles^26^ and make a diagnosis on the proband alone^27^. In Case 5, we identified a VUS in *trans* by genetically phasing the patient’s genome through long-read sequencing that provides a moderate criterion to help reclassify the VUS as likely pathogenic

A recent report investigating VUS in a cohort of IRDs indicated that VUS was more likely to be detected in exonic regions and none were detected in non-coding intronic or upstream promoter regions^11^. However, this work identifies a deep intronic VUS in the *NPHP4* gene in Case 1. In patients who demonstrate clinical features typical for an IRD, and in whom only one pathogenic coding variant has been identified by exome sequencing, the second pathogenic variant in non-coding regions of the gene resulting in hypomorphic gene function^9^ can account for the missing heritability. Non-coding variants can more readily be identified by targeted long-read genome sequencing^16^. This approach is invaluable since one of the variants might be de novo and data from parents is not always available to exclude the possibility that variants reside in *cis*. With the expanding role of non-coding variants accounting for the missing heritability, long-read sequencing can be instrumental in elucidating these complex disease-causing variants^21^.

In summary, in this report, we reclassify 5 VUS in IRDs as likely pathogenic and address methods through which long-read sequencing can alleviate the bottleneck of haplotype analysis using familial co-segregation analysis. Future studies may include using long-read sequencing to analyze and reclassify more VUS in genes associated with AR IRDs without the need for parental testing.

## Methods

### Case selection

This study was approved by the institutional review board at the University of Washington (STUDY00014158). Written informed consent was obtained from all study subjects for a blood draw for genetic testing. Experiments were conducted according to the principles expressed in the Declaration of Helsinki. A retrospective chart review of patients with non-syndromic and syndromic IRDs who underwent NGS with subsequent co-segregation analysis was performed and six individuals were identified for further data collection. All six participants underwent clinical exome-based panel testing for IRDs and their family members underwent targeted variant analysis to further classify any variants of uncertain significance. Cases 1 and 3 underwent testing at the University of Washington Laboratory for Precision Diagnostics (CLIA # 50D0631935) with Case 1 undergoing a clinical whole exome test and Case 3 undergoing a 20 gene Stargardt Disease and other Macular Dystrophies Panel. Cases 2 and 5 both underwent genetic testing through Blueprint Genetics (CLIA # 99D2092375) with the Retinal Dystrophy Panel. Case 3 was subjected to the Retinal Dystrophy Pane from Molecular Vision Labs (CLIA # 38D2059762). Baseline data at the patients’ initial ophthalmology encounter was collected, including patient age, gender, slit lamp and fundoscopic examination findings, imaging results, and clinical diagnosis. Genetic testing results of the subjects and their family members were collected.

### Bioinformatic analysis

The gnomAD database^4^ was used for variant annotation of allelic frequency and variant assessment^23^. Prediction of variant pathogenicity was interpreted from the use of SIFT^28,29^, PolyPhen-2^30^, and CADD^31,32^ whereas prediction of splicing or intronic variants was interpreted using Splice-AI^33^.

### Classification criteria of VUS

Criteria established by the ACMG were applied to classify VUS^22^. This involved combining criteria from population data encompassing the frequency of variants within healthy and diseased populations (gnomAD database), in silico predictive data to determine variant consequence at the nucleotide or amino acid level, haplotype/allelic data (from familial testing), de novo data, allelic data and other relevant ophthalmologic data such as disease features specific for the gene. Various criteria ranging from strong to moderate to supporting categories were utilized to provide evidence to classify VUS in each case.

### Extraction of genomic DNA, long-read library preparation, targeted sequencing enrichment, and sequencing analysis

Informed consent was obtained to carry out a venipuncture blood draw of 2 mL for Case 5. Genomic DNA (gDNA) was isolated using the Qiagen MagAttract High Molecular Weight genomic DNA isolation kit (Qiagen). Approximately 1.2 μg of gDNA was used to make sequencing libraries using the Oxford Nanopore Technologies (ONT) Ligation Sequencing Kit (SQK-LSK110) with slight modifications to the manufacturer’s instructions. The long fragment buffer was used during the clean-up to enrich for fragments >3 kilobases in size. All 15 microliters of the library were loaded onto a 9.4.1 flow cell for sequencing on an ONT MinION running MinKNOW control software with adaptive sampling implementation. Target regions of *USH2A* were enriched using Readfish^34^ adaptive sampling technology implemented during real-time sequencing. Sequencing experiments were run for up to 48–72 h. The *N*_50_ (the read length such that reads of this length or greater sum to at least half the total bases) was 12,455 bases, whereas the longest read spanned >240,000 bases. FASTQ files were generated using Guppy and aligned to GRCh38 using minimap2^35^. Variants were called using PEPPER and haplotyping was achieved using Margin and finally DeepVariant was used to get the alignment file to generate a phased VCF file^36^.

### Reporting summary

Further information on research design is available in the Nature Research Reporting Summary linked to this article.

### Supplementary information

Reporting Summary

## Data Availability

The gnomAD and additional data tools (i.e. CADD) used in this work are publicly accessible. The variant data in this study are included within the published article and have been deposited in ClinVar. Genome sequencing data are not publicly available due to privacy and patient anonymity issues. Access to the genome sequencing data will require an IRB-approved collaboration and Data Usage Agreement.
